# Relationship Between the Menstrual Cycle and Timing of Ovulation Revealed by New Protocols: Analysis of Data from a Self-Tracking Health App

**DOI:** 10.2196/jmir.7468

**Published:** 2017-11-27

**Authors:** Satoshi Sohda, Kenta Suzuki, Ichiro Igari

**Affiliations:** ^1^ Department of Obstetrics and Gynaecology Graduate School of Medical Science University of Tsukuba Tsukuba Japan; ^2^ Biodiversity Conservation Planning Section Center for Environmental Biology and Ecosystem Studies National Institute for Environmental Studies Tsukuba Japan; ^3^ Advanced Technology Section R&D Division MTI Ltd Shinjyuku Japan

**Keywords:** self-tracking, person generated health data, calendar calculation, fertility, menstrual cycle

## Abstract

**Background:**

There are many mobile phone apps aimed at helping women map their ovulation and menstrual cycles and facilitating successful conception (or avoiding pregnancy). These apps usually ask users to input various biological features and have accumulated the menstrual cycle data of a vast number of women.

**Objective:**

The purpose of our study was to clarify how the data obtained from a self-tracking health app for female mobile phone users can be used to improve the accuracy of prediction of the date of next ovulation.

**Methods:**

Using the data of 7043 women who had reliable menstrual and ovulation records out of 8,000,000 users of a mobile phone app of a health care service, we analyzed the relationship between the menstrual cycle length, follicular phase length, and luteal phase length. Then we fitted a linear function to the relationship between the length of the menstrual cycle and timing of ovulation and compared it with the existing calendar-based methods.

**Results:**

The correlation between the length of the menstrual cycle and the length of the follicular phase was stronger than the correlation between the length of the menstrual cycle and the length of the luteal phase, and there was a positive correlation between the lengths of past and future menstrual cycles. A strong positive correlation was also found between the mean length of past cycles and the length of the follicular phase. The correlation between the mean cycle length and the luteal phase length was also statistically significant. In most of the subjects, our method (ie, the calendar-based method based on the optimized function) outperformed the Ogino method of predicting the next ovulation date. Our method also outperformed the ovulation date prediction method that assumes the middle day of a mean menstrual cycle as the date of the next ovulation.

**Conclusions:**

The large number of subjects allowed us to capture the relationships between the lengths of the menstrual cycle, follicular phase, and luteal phase in more detail than previous studies. We then demonstrated how the present calendar methods could be improved by the better grouping of women. This study suggested that even without integrating various biological metrics, the dataset collected by a self-tracking app can be used to develop formulas that predict the ovulation day when the data are aggregated. Because the method that we developed requires data only on the first day of menstruation, it would be the best option for couples during the early stages of their attempt to have a baby or for those who want to avoid the cost associated with other methods. Moreover, the result will be the baseline for more advanced methods that integrate other biological metrics.

## Introduction

Awareness of one’s own fertility is considered important in helping women become pregnant in a shorter period of time [1-3]. Fertility depends on the menstrual (ovarian) cycle, and in each cycle there is a “fertile window” during which women can conceive [1,4,5]. Studies on standard menstrual cycles suggest that the fertile window starts 5 days prior to ovulation and ends on the day of ovulation [4]. This is essentially consistent with the results of statistical studies [1,5] that estimated the day-specific pregnancy rates; the pregnancy rate starts to increase 8 days prior to ovulation, peaks after 6 days (2 days prior to ovulation), and reaches almost 0 at 2-3 days after ovulation. Hence, in order to be aware of the fertility window, it is important for a woman to be able to predict the next ovulation date in the course of her menstrual cycles.

The “calendar method” of predicting the next ovulation date was developed based on the recognition of cycles in the menstrual period and fertility, in which women record their menstrual cycles for family planning [6]. It may have been one of the most widely recorded personal health information details before the spread of mobile phones and computers. On the other hand, the recent popularity of self-tracking tools realized by ubiquitous and wearable technologies has led people to gather various kinds of self-information ranging from financial behaviors to physical activities [7,8]. These technologies are used to “help people collect personally relevant information for the purpose of self-reflection and gaining self-knowledge” and are referred to as personal informatics systems [9]. Currently, the classical calendar method of predicting the next ovulation date is integrated into personal informatics systems. There are many mobile phone apps aimed at helping women map their ovulation and menstrual cycles and facilitating successful conception (or avoiding pregnancy) [10,11]. Apps available for these purposes include Ovia Fertility (Ovuline), Glow, OvuView, Ovulation Calendar, Fertility Calendar, My Days, Period Diary, Period Tracker, Maybe Baby, and Fertility Friend [12]. As Lupton [12] mentioned, while these apps are grounded on traditional gynecological knowledge, the advantage that some of them claim is a data analytic approach that can provide greater accuracy than more traditional forms of self-tracking. These apps usually ask users to input various biological features (eg, ovulation, sexual intercourse, basal body temperature, state of cervical mucus, body weight, and the timing of menstrual bleeding).

However, it is not known how to process these features numerically to improve the ovulation prediction error. A mixture of knowledge on biological mechanisms and a statistical approach using the newly enabled biological metrics is promising [13,14], although it is still an open problem. Instead of pursuing the usage of various biological metrics, we believe that more attention should be paid to other aspects of this dataset, that is, its massiveness. It is a remarkable achievement that mobile phone apps have been able to accumulate menstrual cycle data of a vast number of women. Currently, records of more than 10,000 individuals can be a target of statistical analysis. In this study, we start from calendar-based methods that require only the recording of menstruation to predict the ovulation date [15-17]. For many couples, the calendar-based methods are the simplest options of determining the timing of the menstrual cycle [2]. Even within the simple prediction framework, a large amount of data potentially allows us to figure out individual differences better than in traditional understanding [18,19].

This study aims to clarify the above points using data obtained from a commercial women’s health care service provided as a mobile phone app in Japan. We extracted approximately 0.1% of all users of the app, resulting in 7043 subjects after data screening. This paper reports on the progressive health data ecosystem in which commercial health care mobile apps generate massive amounts of data. The results of the data analysis give feedback to the app and can be used to improve public health as well.

## Methods

### Data

We screened 150,000 users who wanted to conceive out of a total of 8,000,000 users of a mobile phone app from a commercial women’s health care service, Luna Luna. We used the data of 7043 women who had each recorded at least one menstrual cycle with ovulation date, suggesting that about 5% of women utilize ovulation tests to support their conception. The ovulation date had been determined by one of the methods described in the next paragraph. The total number of cycles was 135,666, and there were 12,731 cycles with an ovulation date. Any cycles in the record that were less than 20 days or greater than 45 days were removed to rule out unnatural cycle length that is due to erroneous or defective input. About 57% (7285) of the cycles had more than 8 records of past menstrual cycles after the screening, which allowed us to analyze the relationship between past and future menstrual cycles. The age distribution of the 7043 women ranged from 20-45 years with a mean of 32.94 years (95% CI 32.04-33.85), which is slightly higher than the mean maternal age of Japanese women at the time of first birth (which was 30.1 years in 2010).

Each user’s personal records consisted of the dates when they recognized menstrual bleeding (onset of menstruation) and the dates when they detected ovulation. In the log file, an identifier is attached to each ovulation record to distinguish its basis (clinical diagnosis/ovulation test kit/other reliable method). In our analysis, only clinical diagnosis‒based (31%) and ovulation test kit‒based (54%) ovulation records were used. Luna Luna does not ask women to record which clinical diagnostic test they used to determine the ovulation date. However, it is noted that the ovulation day in Japan is commonly determined by ultrasound scanning and occasionally with testing of blood luteinizing hormone or estrogen level.

The Luna Luna data are the property of MTI Ltd (Shinjyuku, Tokyo, Japan). The authors (KS and II) are employees of MTI Ltd and are permitted to access the Luna Luna data server. The information security committee of MTI Ltd concluded that this study does not require approval by an ethics committee because the data are anonymized appropriately; the data server used in the study is a backup of the original data server, on which anonymous IDs are placed on personally identifiable information. Hence, it was impossible for the authors to access personally identifiable information, which was controlled separately. Consent for data use and information acquisition was obtained from Luna Luna users, as stipulated in the terms of use.

### Luna Luna

Luna Luna is a total health care service for female mobile phone users in Japan. Luna Luna offers its users predictions of menstrual cycles, fertility, ovulation, and related health care information, based on user-inputted personal records that are sent and stored in its data server. The data are securely stored separately from personally identifiable information. Luna Luna has been provided as a commercial service for more than 10 years since the year 2000. Luna Luna has 7 million subscribers as of 2016 and occupies a leading position among mobile health care services for female users in the Japanese market.

### Menstrual Cycles, Timing of Ovulation, Follicular Phases, and Luteal Phases

We express the records of the first day of menstruation of woman *i* as,

*M_i_=(m*_i1_*, m*_i2_*,...,m*_iT_*)*,

where *m*_i1_ is the first day of the most recent menstruation of woman *i (i=1, 2,...,N)*, *m*_i2_ is the first day of her second most recent menstruation, and so on. Then, we defined *Ci* as a series of menstrual cycle lengths of woman *i* by,

*C*_i_*=(c*_i1_*,...,c*_iT–1_*)=(D(m*_i1_*, m*_i2_*),...,D(m*_iT–1_*, m*_iT_*))*,

where *c*_ij_ denotes the *j* th most recent menstrual cycle of woman *i* and *D(m*_ij_*, m*_ij+1_*)* denotes a function that gives the number of elapsed days between *m*_ij+1_ and *m*_ij_. For simplicity of notation, we define the mean length of the menstrual cycles of woman *i* over the *j* th to *j’* th cycles as *c*_i_*(j, j’)=Σ_t=j,...,j’_*c*_it_*/(j’–j+1).*

*F*_i_ is the series of follicular phase lengths of woman *i*, where each follicular phase length, *f*_ij_, is defined as follows: assuming we have records of ovulation *o*_ij_*(j=1, 2, ...)* between *m*_ij_ and *m*_ij+1_,

*F*_i_*=(..., f*_ij_*, ...)=(..., D(o*_ij_*, m*_ij+1_*), ...)*.

Thus, the timing of ovulation is *f*_ij_ days after the day of the previous menstruation *m*_ij+1_. Similarly, the series of luteal phase lengths of woman *i*, *L*_i_, where each luteal phase length is *l*_ij_, is defined as,

*L*_i_*=(..., l*_ij_*, ...)=(..., D(m*_ij_*–1, o*_ij_*+1), …)*.

Here, *o*_ij_*+1* is the day after ovulation and *m*_ij_*–1* is the day before the next menstruation. The timing of ovulation is *c*_ij_*–l*_ij_*–1* days after the first day of the previous menstruation *m*_ij+1_. It should be mentioned that the length of records varied among the women. We used *T*_i_ to indicate the length of records of woman *i*.

We investigated the relationships between the length of menstrual cycles and the length of the follicular phases or that of the luteal phases. We also analyzed the relationships between the mean length of past menstrual cycles and the length of the follicular phases or that of the luteal phases because prediction of ovulation date requires an unknown length of the next menstrual cycle.

### Calendar Calculations

We evaluated the relevance of three calendar-based methods using our data. The first was the Ogino method [15], which assumes a fixed length of the luteal phase of 14 days and predicts the ovulation date as [c*]–15 days after the onset of the previous menstruation for a particular woman when the mean length of her menstrual cycles is c* (here [c*] represents c* rounded down to the nearest integer). This implies that the length of the follicular phase in the next cycle is [c*]–15. The Ogino method is the most widespread calendar-based method relied on by Japanese women. The second method is the method proposed by Lamprecht and Grummer-Strawn [16], which assumes that the length of the next follicular phase is [c*/2], and thus this method predicts a woman’s next ovulation date as [c*/2] days after the onset of the previous menstruation. Here, we call this method as the half cycle length (HCL) method. Because of its simplicity, we chose the HCL method over other calendar-based methods that reflect individual differences in the length of the luteal phase in a menstrual cycle. The third method is the method that was developed in this study and predicts a woman’s next ovulation date as *f*_i_(c*) days after the onset of the previous menstruation. As explained in the next section, *f*_i_ is a linear function that is optimized by using the relationship between the follicular phase length and *c* *. Hence, we call the third method as the Optimized method.

To analyze the relationship between the timing of ovulation and the mean length of past menstrual cycles, we evaluated the three prediction models for follicular phase length, that is, μ_Ogino_, μ_HCL_, and μ_OPT_, which predict the length of the next follicular phase as *μ*_Ogino_*(C*_i_*, j, k)=[c*_ij_*(k)]–15, *μ*_HCL_*(C*_i_*, j, k)=[c*_ij_*(k)/2], and *μ*_OPT_*(C*_i_*, j, k)=[f*_ij_*(c*_ij_*(k))], respectively. Here, *c*_ij_*(k) is defined as *c*_ij_*(k)=c_i_*(j+1. J+k). It should be noted that if *k=1*, *c*_ij_*(k) is identical to the nearest cycle length, *c*_ij+1_.

To predict the timing of ovulation, we used the results of least square fitting between mean cycle length and follicular phase length. The prediction performance of the timing of ovulation using the obtained model, *μ*_OPT_, and the prediction performances using the Ogino and HCL methods, *μ*_Ogino_ and *μ*_HCL_, respectively, were compared with different allowable prediction error levels, *|μ–f*_ij_*| ≤ 0, 1, 2*.

### Linear Models

In our analysis, we used a linear model to describe the relationship between an explanatory variable *x* and a response variable *y*. For data that consist of multiple data points from each individual, linear models are generally categorized into two types: fixed effect models and random effect models [20]. A fixed effect model is formalized as follows:

*y*_ij_*=α*_1_*δ*_i1_*+...+α*_n_*δ*_in_*+x*_ij_*β+ϵ*_ij_,

*δ*_ij_*=1* if *i=j* else *0*,

where *δ*_ij_ s are dummy variables. The least square estimate of parameters including dummy variables is obtained as,

*β*_lsdv_*=∑*_i=1,...,N_*∑*_t=1,...,Ti_*(x*_ij_*–x*_i_*)(x_ij_*–x*_i_*)/∑_i=1,...,N_*∑*_t=1,...,Ti_*(x*_ij_*–x*_i_*)(y_ij_*–y*_i_*),

*α*_i_*=y*_i_*–x_i_*β_lsdv_,

where *x*_i_*=1/T_i_*∑*_t=1,...,Ti_*x*_it_ and *y*_i_*=1/T_i_*∑*_t=1,...,Ti_*y*_it_. Hence, in this model, different women have different *α*_i_ for the relationship between *x* and *y*, and there is a correlation between *α* and x. On the other hand, in random effect models, the random component of *α*_i_ s is included in the random variable *μ*_ij_ as follows:

*y*_ij_*=α+ x*_ij_*β+μ*_ij_=*x*_ij_*’β’+μ*_ij_,

Here, we assumed that x_ij_'=(1, x_ij_) and β'=(α, β). A pooled ordinary least square estimate, β_p_'=(α_p_, β_p_), is obtained as,

*β*_p_*’=∑*_i=1,...,N_*∑*_t=1,...,Ti_*x*_it_*’y*_it_*/∑*_i=1,...,N_*∑*_t=1,...,Ti_*x_it_’*^2^.

However, the result underestimates the covariance structure in *μ*_ij_ s, which is described as a matrix,

*Ω={ω*_ij_*}*_i, j=1,...,N_,

*ω*_ij_*=ξ*_α_^2^*+ξ*_ϵ_^2^ if *i=j* else *ξ*_α_^2^.

Using the result of pooled ordinary least square, the generalized least square estimate of parameters that include the effect of covariance, *β*_gls_*'=(α*_gls_*, β*_gls_*)*, is obtained as,

*β*_gls_*’=∑*_i=1,...,N_*∑*_t=1,...,Ti_*∑*_s=1,...,Ti_*ω*_ts_^(–1)^*x*_it_*’y*_it_*/∑*_i=1,...,N_*∑*_t=1,...,Ti_*∑*_s=1,...,Ti_*ω*_ts_^(–1)^*x*_it_*’*^2^*,*

where *ω*_ts_^(–1)^ is an element of *Ω*^(–1)^, which is the inverse matrix of *Ω*. To obtain *ξ*_α_ and *ξ*_ϵ_, we first calculate,

*ξ*_μ_*=1/(NT–p)∑*_i=1,...,N_*∑*_t=1,...,Ti_*μ*_it_*,*

and

*ξ*_α_*=1/{NT(T–1)/2–p}∑*_i=1,...,N_*∑*_t=1,...,Ti–1_*∑*_s=t+1,...,Ti_*μ*_it_*μ*_is_*,*

then,

ξ_ϵ_=ξ_μ_–ξ_α_

In this paper, we used the Hausman test to determine which of the models better explains the data. In the Hausman test, the percentile value of *H=(β*_gls_*–β*_lsdv_*)*^2^ in a *Χ*^2^ distribution with one degree of freedom is calculated, and a random effect model is rejected if the *P* value is greater than .05.

## Results

In our dataset, the mean (95% confidence interval) of the menstrual cycle length, the follicular phase length, and the luteal phase length of the 7043 women over all cycles was 29.76 (24-38), 14.84 (10-23), and 13.91 (10-19) days, respectively. Both the length of the follicular phases and of the luteal phases had a positive correlation with the length of the menstrual cycles (Table 1). The Pearson correlation coefficient between the length of the menstrual cycles and the length of the follicular phases or of the luteal phases was .75 (*P*<.001) and .37 (*P*<.001), respectively. Hence, both the follicular phase length and luteal phase length had significant positive correlations with the menstrual cycle length. For analysis of the relationship between the length of the menstrual cycles and the length of the follicular phases or of the luteal phases, we applied the random effect model because the *P* value of the Hausman test was .001. The coefficient (α, β) of the generalized least square estimate was (.501, -.088) for the follicular phase length and (.466, .088) for the luteal phase length.

We then investigated the relationship between the mean length of past cycles and the cycle length, follicular phase length, and luteal phase length of the next menstrual cycle. Table 2 shows the number of cycles having enough records to calculate *c*_ij_*(k) for each number of k. For example, there were 11,640 cycles with at least one previous cycle, and there were 7285 cycles having records of 8 past cycles. Table 3 shows the Pearson correlation coefficients between *c*_ij_*(k) and the next menstrual cycle length, follicular phase length, and luteal phase length. Both the next cycle length and the follicular phase length had strong correlations with the mean cycle length. Only a weak correlation was found between the mean cycle length and the luteal phase length, although it was statistically significant. We applied the random effect model because the *P* value of the Hausman test was <.050 for all cases (Table 4). The coefficient (α, β) of the generalized least square estimate was similar to that calculated for the actual cycle length. In summary, the menstrual cycle length had positive correlations with both the follicular phase length and luteal phase length, although the correlation was less strong with luteal phase length.

**Figure 1:**
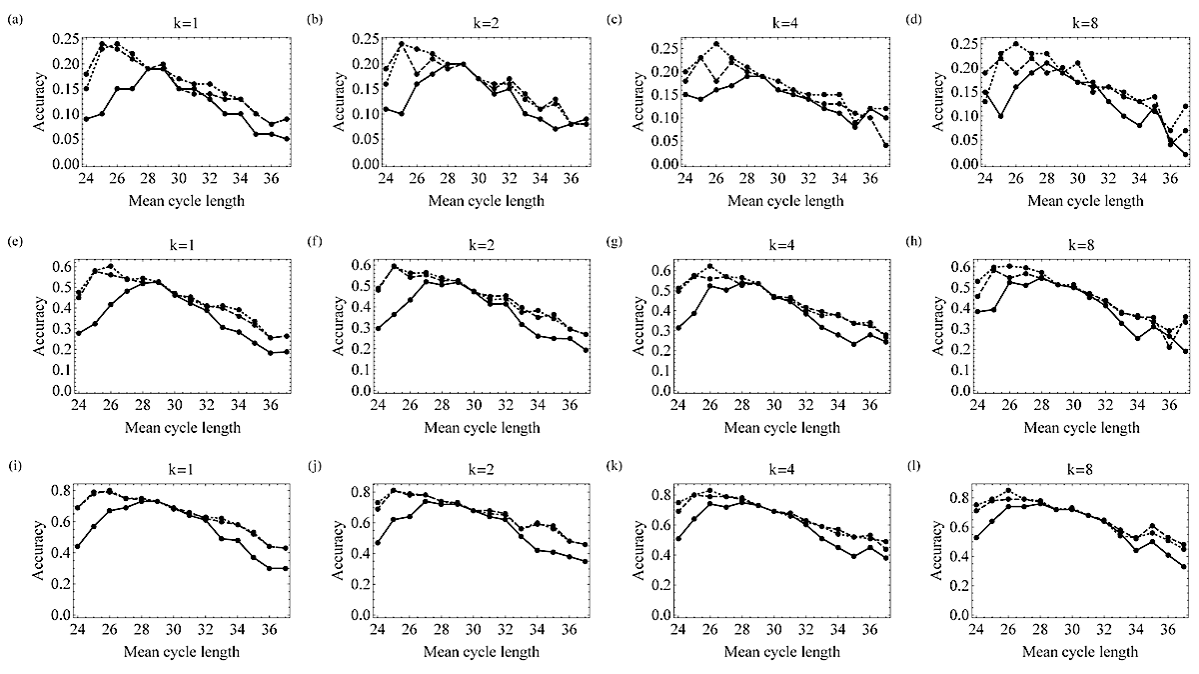
Accuracy of the Ogino, HCL, and the Optimized methods in predicting the day of the next ovulation date with allowable prediction error=0 (a-d), 1 (e-h), and 2 (i-j) for different mean cycle lengths (solid line, dashed line, and dotted line indicate the Ogino, HCL, and the Optimized methods, respectively).

**Figure 2:**
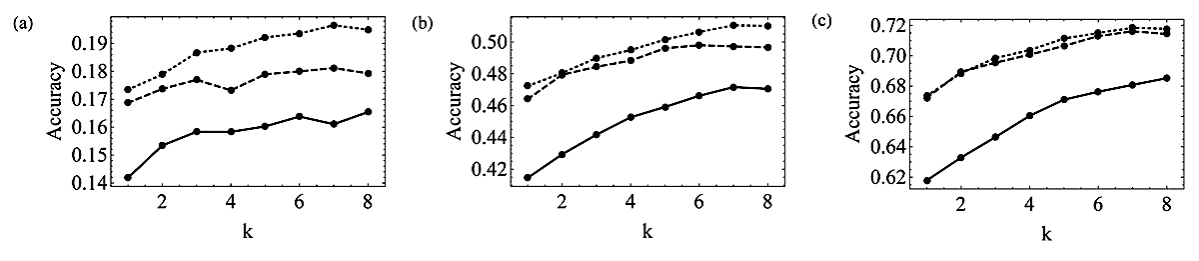
Mean accuracy over different mean cycle lengths with allowable prediction error=0 (a), 1 (b), and 2 (c) (solid line, dashed line, and dotted line indicate the Ogino, HCL, and the Optimized methods, respectively).

The prediction performance of the timing of ovulation using the obtained model, *μ*_OPT_, was compared with that of *μ*_Ogino_ and *μ*_HCL_. *μ*_OPT_ outperformed *μ*_Ogino_ when a woman’s mean cycle length was shorter than 27 days or longer than 31 days (Figure 1). *μ*_OPT_ outperformed *μ*_HCL_ when a woman’s mean cycle length was less than 28 days. As for the mean accuracy over different mean cycle lengths (Figure 2), *μ*_OPT_ outperformed *μ*_Ogino_ in all cases. The prediction performances of *μ*_OPT_ and *μ*_HCL_ were similar when only small numbers of cycles were available to calculate the mean cycle length, whereas *μ*_OPT_ showed an advantage with increasing values of *k*.

**Table 1:** Relationship between cycle length, follicular phase length, and luteal phase length.

Cycle length, days	Cycles, n	Mean follicular phase length, days	95% CI	Mean luteal phase length, days	95% CI
23	120	10.5	(7-15)	11.5	(7-15)
24	324	11.1	(7-15)	11.9	(8-16)
25	657	11.5	(8-16)	12.5	(8-16)
26	1065	12.1	(9-16)	12.9	(9-16)
27	1407	12.7	(10-16)	13.3	(10-16)
28	1637	13.4	(10-17)	13.6	(10-17)
29	1516	14.1	(11-17)	13.9	(11-17)
30	1392	15	(11-19)	14	(10-18)
31	1144	15.7	(12-20)	14.3	(10-18)
32	875	16.4	(12-20)	14.6	(11-19)
33	707	17.4	(13-21)	14.6	(11-19)
34	557	18.1	(12-23)	14.9	(10-21)
35	395	18.8	(11-26)	15.2	(8-23)
36	300	19.6	(12-25)	15.4	(10-23)
37	204	20.1	(12-25)	15.9	(11-24)
38	162	21.5	(14-28)	15.5	(9-23)
39	145	21.6	(12-29)	16.4	(9-26)
40	124	22.3	(12-29)	16.7	(10-27)

**Table 2:** Number of cycles having records of k past cycles.

Mean cycle length, days	k=1	2	3	4	5	6	7	8
24	176	168	117	121	90	91	67	68
25	438	496	384	385	325	313	282	272
26	878	918	805	798	694	700	625	608
27	1253	1291	1194	1222	1129	1083	1003	911
28	1569	1529	1507	1440	1382	1293	1196	1154
29	1620	1541	1472	1361	1306	1270	1178	1078
30	1396	1328	1292	1238	1181	1074	998	934
31	1101	1024	1036	979	964	895	860	785
32	862	856	867	728	715	647	624	560
33	721	637	564	569	520	463	429	378
34	532	448	461	371	362	301	276	225
35	377	353	314	253	226	188	195	174
36	290	214	194	179	141	110	88	76
37	219	160	125	90	74	65	47	42
Total	11,640	11093	10421	9804	9154	8529	7898	7285

**Table 3:** Pearson correlation coefficients between mean cycle length and next cycle length, follicular phase length, or luteal phase length (*P*<.001 for all cases).

	k=1	2	3	4	5	6	7	8
Next cycle length, days	.543	.574	.584	.589	.589	.593	.592	.592
Follicular phase length, days	.506	.526	.537	.547	.547	.546	.548	.547
Luteal phase length, days	.109	.129	.138	.135	.137	.144	.140	.140

**Table 4:** Results of least square fitting and the Hausman test.

		k=1	2	3	4	5	6	7	8
**Follicular phase length**									
	Α	.528	.528	.527	.526	.526	.525	.525	.523
	Β	.039	.017	.012	.003	–.002	–.010	–.011	–.011
	*P* value	.001	.001	.001	.001	.001	.001	.001	.001
**Luteal phase length**									
	Α	.465	.467	.470	.471	.471	.472	.472	.473
	Β	.039	.017	.012	.003	–.002	–.010	–.011	–.011
	*P* value	.001	.001	.001	.001	.001	.001	.001	.001

## Discussion

### Principal Findings

In this paper, using the data obtained from the Luna Luna service, we evaluated how menstrual cycle length is related to luteal phase length and follicular phase length. As suggested in a previous study [21], the correlation between length of menstrual cycles and length of follicular phases was stronger than the correlation between length of menstrual cycles and length of luteal phases (Table 1). Furthermore, there was a positive correlation between the lengths of past and future menstrual cycles (Tables 3 and 4); this suggests that there is regularity in menstrual cycles as claimed by Creinin et al [22]. A strong positive correlation was also found between the mean length of past cycles and the mean length of follicular phases. Thus, the follicular phase length can be mostly estimated from each woman’s menstrual cycle length. However, the correlation between mean cycle length and luteal phase length was also statistically significant. Our results showed that the random effect model was relevant for modeling the relationship between menstrual cycle length and follicular phase length as well as the relationship between menstrual cycle length and luteal phase length. Furthermore, the random effect model was also relevant for modeling the relationships between mean length of past menstrual cycles and mean length of follicular phases or luteal phases. These results suggest that these relationships are shared among all women without being influenced by personal factors. The existing calendar-based methods (Ogino and HCL methods) did not explain our data well. Against the assumption of the Ogino method, the weak positive correlation between mean cycle length and luteal phase length (Tables 3 and 4) suggests that the luteal phase does not have a constant length among women with mean menstrual cycles of different lengths. This is why the Ogino method was less accurate when a woman’s mean cycle length was shorter than 27 days or longer than 32 days (Figure 1). The HCL method was less accurate when a woman’s mean cycle length was less than 28 days (Figure 1). By taking the average over different mean cycle lengths, the accuracy of the Ogino method was worse than that of the other methods as well (Figure 2). The accuracy of the HCL method was close to that of the Optimized method when there were only a small number of cycles available to calculate the mean cycle length (Figure 2). The Optimized method showed better performance with increasing numbers of available cycles. The Optimized method outperformed the HCL method in explaining the relationship between the mean cycle length and the follicular phase length when a large number of past cycles were available to calculate the mean cycle length. These results showed that the performance of the Optimized method was equivalent to or better than that of the HCL method. Hence, we recommend using the Optimized method to predict the timing of ovulation from the mean length of menstrual cycles if these data are available.

The novelty of our findings is essentially accounted for by the large number of participants. The Ogino method was developed based on the anatomical observation of ovarian follicles of 81 women with cycle lengths of between 23 and 45 days [15]. They reported that there was a variable luteal phase length (12-16 days), while the number of subjects studied was not large enough to identify the more detailed relationship between the lengths of the menstrual cycle, follicular phase, and luteal phase. Three studies with more data reported the mean (95% CI) of the follicular phase length and the luteal phase length as 16.5 (9-23) and 12.4 (8-17) [21], 15.5 (9-22) and 12.6 (9-17) [23], and 15.0 (2-21) and 13.5 (7-20) days [24], respectively. These studies reported only the mean value over all women, except for Fehring et al [21] who reported positive correlations between menstrual cycle length and follicular phase length or luteal phase length. However, their analysis was based on only 1060 cycles in 141 women. Lamprecht and Grummer-Strawn [16] suggested that follicular phase length was better estimated by *c* */2 based on an analysis of 7514 cycles in 1062 women. In contrast, our study analyzed more than 65,000 cycles, which is 61 and 8.5 times greater than what was used in the studies of Fehring et al [21] and Lamprecht and Grummer-Strawn [16], respectively. This study allowed us to capture the relationships between length of menstrual cycle, follicular phase, and luteal phase in more detail.

Recently, mobile health information technology—known as “digital health,” “eHealth,” or “mHealth”—has been used in medicine and public health in various ways [25-28]. With the recent spread of smartphones and other mobile devices (eg, 77% of US adults [29], 62.6% of Japanese [30], and 62% of Chinese [31] own smartphones), digital health technologies have begun to be adapted for a wide variety of purposes. The mHealth information technologies are one of the origins of the advanced movement called “Quantified Self”, which stresses the role of patients or consumers in medicine and public health [18,19,32]. Quantified-self refers to an individual who is engaged in the self-tracking of any kind of biological, physical, behavioral, or environmental information [19]. These movements are now spreading among people who were not familiar with such technologies before [7]. For example, 60% of US adults are currently tracking their weight, diet, or exercise routine, and 33% are monitoring other factors such as blood sugar, blood pressure, headaches, or sleep patterns [33]. There are 165,000 mobile phone health apps available on the market [34]. The self-tracked health data are regarded as the key to realizing personalized medicine and health maintenance [19].

On the other hand, there are several concerns about these technologies. First, Lupton [35] argued that they might allow our personal health data be used as “both an object of surveillance and persuasion” [35]. The author in particular claimed that the sharing of health data on social networking services (SNSs) would result in “public surveillance” where everyone would be a subject of surveillance by others. However, it is less beneficial for women to share their menstrual cycle records on SNSs because menstrual cycles strongly depend on biological and physiological factors. Furthermore, issues about menstrual cycles are “sensitive” personal information that would not likely be a topic of conversation among friends. Hence, it is unlikely that women would start sharing information on their menstrual cycles on SNSs. On the other hand, health informatics systems can integrate these data both systematically and anonymously and provide feedback knowledge at a scale that is not achieved by any person-to-person communications. These systems allow women to maximize the benefit of sharing data on their menstrual cycles (or other sensitive health information) without publicizing the data themselves. Sharon [36] argued that self-tracking health technologies reduce phenomena to numbers and “that this simultaneously displaces other, non-quantifiable yet highly insightful means of knowing and expression”. For example, a small percentage of women are aware that they have somatic symptoms around the time of ovulation, for example, ovulation pain. This self-awareness would help women recognize the timing of ovulation. However, not all women have or are aware of these symptoms. Hence, there is a benefit of summarizing regularity behind menstrual cycles in a simple rule and sharing it as social knowledge [17]. We claim that mHealth technology puts this knowledge sharing onto a new stage because it can relax the restriction that the rules must be simple enough to be handled by anyone. Of course, there is loss of information in the rule extraction process. The providers of health informatics systems should take this point seriously and should aim at designing their systems [7-9] so that users can maintain an appropriate distance between their body and its data representation [35,37]. For this purpose, the interaction between a system and its users should be studied in terms of user behavior to find a better way of presenting predictive performance, and these insights should be incorporated into the design of the system [37-39]. Most people are still sensitive about unintended use of data by for-profit companies. In a recent survey on attitudes toward personal health care data [40], more than 80% of the respondents answered that they were willing to share health and medical information with a personal physician or health care provider (88%) and with nonprofit research organizations (84%). Only 24% answered that they were willing to share their data with for-profit companies. Nevertheless, it is worth noting that 88% answered that their motivation for sharing health information was to make new health discoveries. With increasing numbers of people contributing their health data to analyzable datasets, health information data will not only empower companies and consumers but also benefit health and social systems [18].

### Limitations

Our analysis lacked complete profile data for all subjects and the dataset had inevitable selection bias. Moreover, menstrual bleeding started in the middle of the night in some cases—the date taken as the menstrual start day depended on the user’s subjective choice. However, we believe that having a large-scale dataset available to carry out investigations on women’s health overcomes such limitations.

### Conclusions

Our study demonstrated how the present calendar methods of predicting the ovulation date were improved by the better grouping of women, which can be supported statistically only with massive numbers of subjects. Because calendar methods require only the timing of menstruation, one of the calendar methods would be the best option for couples during the early stages of their attempt to have a baby or for those who want to avoid the cost associated with other methods. Moreover, the results of the calendar method will be the baseline for more advanced methods that integrate other biological metrics. The mobile phone‒based health care services are very efficient in obtaining large datasets because they offer easy ways for users to input and manage their personal data. This recently enabled data collection framework is complementary to existing well-controlled experimental methods and will contribute to the testing of medical hypotheses that previously could not be studied due to insufficient numbers of subjects. Users benefit from newly developed medical knowledge by using mobile phone‒based services without the need to learn intricate calculations. For medical personnel and researchers, the records accumulated by these commercial services can be a useful source of data for analysis after appropriate anonymity processing. Thus, in the mobile phone‒based services that aim at facilitating conception (or contraception), medical specialists and users form a knowledge-improving cycle that can provide quick feedback to the users from the emerging analysis results. Such systems, including other mobile phone health care services, are strongly expected to contribute to comprehensive health care for people of all ages.

## Abbreviations

HCL: half cycle length
OPT: optimal
SNS: social networking service

## References

[1] Dunson DB, Colombo B, Baird DD (2002). Changes with age in the level and duration of fertility in the menstrual cycle. Hum Reprod.

[2] Stanford JB, White GL, Hatasaka H (2002). Timing intercourse to achieve pregnancy: current evidence. Obstet Gynecol.

[3] Wilcox AJ, Weinberg CR, Baird DD (1995). Timing of sexual intercourse in relation to ovulation. Effects on the probability of conception, survival of the pregnancy, and sex of the baby. N Engl J Med.

[4] Mihm M, Gangooly S, Muttukrishna S (2011). The normal menstrual cycle in women. Anim Reprod Sci.

[5] Colombo B, Masarotto G (2000). Daily Fecundability. DemRes.

[6] Spieler JM, Collins WP (2001). Potential fertility - defining the window of opportunity. J Intern Med Res.

[7] Rapp A, Cena F (2016). Personal informatics for everyday life: How users without prior self-tracking experience engage with personal data. International Journal of Human-Computer Studies.

[8] Epstein DA, Ping A, Fogarty J, Munson SA (2015). A lived informatics model of personal informatics.

[9] Li I, Dey A, Forlizzi J (2010). A stage-based model of personal informatics systems. https://pdfs.semanticscholar.org/f90d/ffcab9850a03a4733abb31c123f244ff5f6c.pdf.

[10] Simmons RG, Shattuck DC, Jennings VH (2017). Assessing the Efficacy of an App-Based Method of Family Planning: The Dot Study Protocol. JMIR Res Protoc.

[11] Mangone ER, Lebrun V, Muessig KE (2016). Mobile Phone Apps for the Prevention of Unintended Pregnancy: A Systematic Review and Content Analysis. JMIR Mhealth Uhealth.

[12] Lupton D (2015). Quantified sex: a critical analysis of sexual and reproductive self-tracking using apps. Cult Health Sex.

[13] Pallone SR, Bergus GR (2009). Fertility awareness-based methods: another option for family planning. J Am Board Fam Med.

[14] Fehring RJ (2005). New low- and high-tech calendar methods of family planning. J Midwifery Womens Health.

[15] Ogino K (1932). Uber den konzeptionstermin des weibes und seine anwendung in der praxis. Zentralbl Gynakol.

[16] Lamprecht VM, Grummer-Strawn L (1996). Development of new formulas to identify the fertile time of the menstrual cycle. Contraception.

[17] Arévalo M, Jennings V, Sinai I (2002). Efficacy of a new method of family planning: the Standard Days Method. Contraception.

[18] Swan M (2012). Health 2050: The realization of personalized medicine through crowdsourcing, the quantified self, and the participatory biocitizen. Journal of Personalized Medicine.

[19] Swan M (2013). The quantified self: Fundamental disruption in big data science and biological discovery. Big Data.

[20] Schmidheiny K, Basel U (2011). Panel data: fixed and random effects. Short Guides to Microeconometrics.

[21] Fehring R, Schneider M, Raviele K (2006). Variability in the phases of the menstrual cycle. J Obstet Gynecol Neonatal Nurs.

[22] Creinin MD, Keverline S, Meyn LA (2004). How regular is regular? An analysis of menstrual cycle regularity. Contraception.

[23] France JT, Graham FM, Gosling L, Hair P, Knox BS (1991). Characteristics of natural conceptual cycles occurring in a prospective study of sex preselection: fertility awareness symptoms, hormone levels, sperm survival, and pregnancy outcome. International Journal of Fertility.

[24] World Health Organization (1984). A prospective multicentre study of the ovulation method of natural family planning. IV. The outcome of pregnancy. World Health Organization. Fertil Steril.

[25] Fiordelli M, Diviani N, Schulz PJ (2013). Mapping mHealth research: a decade of evolution. J Med Internet Res.

[26] Free C, Phillips G, Galli L, Watson L, Felix L, Edwards P, Patel V, Haines A (2013). The effectiveness of mobile-health technology-based health behaviour change or disease management interventions for health care consumers: a systematic review. PLoS Med.

[27] Klasnja P, Pratt W (2012). Healthcare in the pocket: mapping the space of mobile-phone health interventions. J Biomed Inform.

[28] Boulos MN, Wheeler S, Tavares C, Jones R (2011). How smartphones are changing the face of mobile and participatory healthcare: an overview, with example from eCAALYX. Biomed Eng Online.

[29] Pew Research Center Device ownership over time.

[30] Economic Research Office, General Policy Division, Information and Communications Policy Bureau (2015). White Paper. Information and Communications in Japan.

[31] Hsu J, Liu D, Yu Y, Zhao HT, Chen ZR, Li J, Chen W (2016). The Top Chinese Mobile Health Apps: A Systematic Investigation. J Med Internet Res.

[32] Swan M (2009). Emerging patient-driven health care models: an examination of health social networks, consumer personalized medicine and quantified self-tracking. Int J Environ Res Public Health.

[33] Fox S, Duggan M (2012). Mobile health 2012.

[34] IMS Institute for Healthcare Informatics.

[35] Lupton D (2012). M-health and health promotion: The digital cyborg and surveillance society. Soc Theory Health.

[36] Sharon T (2016). Self-Tracking for Health and the Quantified Self: Re-Articulating Autonomy, Solidarity, and Authenticity in an Age of Personalized Healthcare. Philos Technol.

[37] Schüll ND (2016). Data for life: Wearable technology and the design of self-care. BioSocieties.

[38] Bentley F, Tollmar K, Stephenson P, Levy L, Jones B, Robertson S, Price E, Catrambone R, Wilson J (2013). Health Mashups. ACM Trans Comput-Hum Interact.

[39] Rapp A, Tirassa M (2017). Know Thyself: A Theory of the Self for Personal Informatics. Human–Computer Interaction.

[40] Pickard K, Swan M (2014). Big desire to share big health data: A shift in consumer attitudes toward personal health information. https://www.aaai.org/ocs/index.php/SSS/SSS14/paper/viewFile/7765/7783.

